# Meet–Test–Treat for HCV management: patients’ and clinicians’ preferences in hospital and drug addiction services in Italy

**DOI:** 10.1186/s12879-021-06983-y

**Published:** 2022-01-04

**Authors:** Massimo Andreoni, Nicola Coppola, Antonio Craxì, Stefano Fagiuoli, Ivan Gardini, Alessandra Mangia, Felice Alfonso Nava, Patrizio Pasqualetti

**Affiliations:** 1 Infectious Diseases, Polyclinic of Rome Tor Vergata, Rome, Italy; 2 Infectious Diseases Unit, Department of Mental Health and Public Medicine, University of Campania Luigi Vanvitelli, Naples, Italy; 3 Department of Gastroenterology, University of Palermo, Palermo, Italy; 4 Gastroenterology Hepatology and Transplantation, ASST Papa Giovanni XXIII, Bergamo, Italy; 5 EpaC Onlus, Italian Liver Patient Association, Monza (MB), Italy; 6 Liver Unit, Casa Sollievo della Sofferenza Hospital, San Giovanni Rotondo, FG Italy; 7 Penitentiary Medicine and Drug Abuse Unit, Public Health Service, Padua, Italy; 8 Section of Medical Statistics, Department of Public Health and Infectious Diseases, Sapienza University of Rome, Piazzale Aldo Moro 5, 00185 Rome, Italy

**Keywords:** HCV, Meet–Test–Treat, Healthcare services, Point of care, Preferences, Conjoint analysis

## Abstract

**Background:**

It has been estimated that the incidence of chronic hepatitis C virus (HCV) will not decline over the next 10 years despite the improved efficacy of antiviral therapy because most patients remain undiagnosed and/or untreated. This study aimed to investigate the opinion of relevant target populations on the practicability, effectiveness and best modalities of the test-and-treat approach in the fight against HCV in Italy.

**Methods:**

A survey was delivered to patients with HCV from the general population, patients from drug addiction services, hospital physicians and healthcare providers for drug addiction services.

**Results:**

For both hospital clinicians and SerD HCPs, tolerability is shown as the most important feature of a suitable treatment. Time to treatment (the time from first contact to initiation of treatment) is deemed important to the success of the strategy by all actors. While a tolerable treatment was the main characteristic in a preferred care pathway for general patients, subjects from drug addiction services indicated that a complete Meet–Test–Treat pathway is delivered within the habitual care center as a main preference. This is also important for SerD HCPs who are a strong reference for their patients; hospital clinicians were less aware of the importance of the patient-HCP relationship in this process.

**Conclusion:**

The health system is bound to implement suitable pathways to facilitate HCV eradication. A Meet–Test–Treat program within the drug addiction services may provide good compliance from subjects mainly concerned with virus transmission.

**Supplementary Information:**

The online version contains supplementary material available at 10.1186/s12879-021-06983-y.

## Introduction

Approximately 71 million people are infected with hepatitis C virus (HCV) worldwide; Italy has the highest number of HCV-positive patients in Europe and the highest death rate from its complications, such as cirrhosis and hepatocellular carcinoma [1, 2]. In Italy, while a first wave between the 1950s and 1960s was associated with unsafe healthcare procedures, the second wave of HCV occurred in the 1980s among people who inject drugs (PWID), reaching a prevalence of > 60% in this population in the 1990s [3]. In 2016, the World Health Organization (WHO) set the goal to reduce the incidence of HCV by 80% and to treat 80% of eligible patients with HCV by 2030 [4–6]. Although traditional primary prevention measures, such as opioid substitution treatment and promotion of safe needle and syringe use, may be effective at preventing HCV transmission, treatment of HCV may be currently proposed as an effective strategy for the prevention of spreading HCV, after the improvement was associated with the introduction of direct-acting antivirals (DAAs), which has made HCV treatment finite and curative [7].

Nevertheless, it is estimated that the incidence of chronic HCV will not decline over the next 10 years despite the improved efficacy of antiviral therapy because most patients remain undiagnosed and/or untreated [8]. This makes identification of at-risk subjects and improvement of access to treatment as mandatory objectives.

The considerable increasing prevalence of HCV in PWID during the last decades showed that unsafe injecting drug use is one of the main contributors to the spread of HCV in Europe [9, 10]. This population is considered difficult to reach for both HCV testing and treatment [11, 12]. For example, an Australian study observed that only 31% of PWID with active or chronic HCV infection that had been previously treated had received specialist HCV assessment, only 8% had received antiviral treatment, and 3% had been cured [13]. In Italy, which is an HCV endemic country, a study of real-life data of diagnosed and treated patients calculated that the eligible pool of patients to treat will run out between 2025 and 2028, depending on the level of linkage-to-care [14].

Increased case finding in targeted, high prevalence groups are required to maintain the treatment rate and achieve the HCV elimination goal.

The test-and-treat model was first proposed by Granich et al. as an intervention strategy in which the at-risk population is screened for HIV infection and individuals diagnosed as infected receive early treatment, aiming to reduce the rate of spreading the virus to other people [15]. This approach has resulted in conceptualizing a landmark of global health policy in being extended beyond HIV infection, and has been proposed for HCV infection [16]. In addition, it may be integrated with point of care testing, facilitating treatment in the habitual care setting, when loss to follow-up and poor adherence to treatment are major impediments for a successful program [17]. A deterministic dynamic compartmental model simulating the impact of test-and-treat and risk-reduction strategies on the HCV epidemic among at-risk people living in France showed that HCV prevalence would decrease from 2.79% in 2015 to 0.48% in 2030 (vs 0.71% with current practices) [16].

To efficiently implement a test-and-treat approach, for which National guidelines are lacking, the needs, opinions and attitudes of all actors in the program must be known and taken into account within the local health system organization. In Italy, two relevant clinical settings can be identified, hospitals and public drug addiction services (SerD), representing two types of patients infected with HCV differently approaching the health system.

This study aimed to investigate the opinion of relevant target populations on the practicability, effectiveness and best modalities of the test-and-treat approach in the fight against HCV, in Italy. Aa discrete choice experiment (DCE) survey was carried out to use a rapid and direct method, and results were evaluated to measure differences of preferences. Among methods to assess preferences, some are more general, such as ranking or rating, and others more specific, including Standard Gamble, Time-Trade-Off, Visual Analogue Scale, Multi-Attribute Utility Instrument and Discrete choice experiment. The last one was chosen since it allows the simultaneous assessment of multiple attributes and is relatively simple since preferences are inferred from a series of stated choices between options, rather than explicitly identified by respondents and, in addition, respondents are not required to quantify their strength of preference for any treatment. The online questionnaire was directed to clinicians dealing with HCV infection, HCV patients, health operators working in SerDs, and subjects followed-up in SerDs.

## Methods

### Study population

An online DCE survey was designed to collect data from two clinical settings in Italy: clinical centers where patients are diagnosed and treated for HCV infection (hospitals) and public health services dedicated to the assistance of addicted subjects (SerD), mostly to IVDU. Four target populations were invited to answer the survey: physicians working in hospitals (hospital clinicians), healthcare providers (HCPs) active in SerDs (SerD HCPs), patients currently or previously infected with HCV and followed-up in hospitals (hospital patients), and addicted subjects followed-up by SerDs (SerD patients).

A list of all Italian hospitals with units devoted to test and treat HCV patients was collected and checked by the project’s organizing secretariat, and an e-mail invitation was sent to them all. Similarly, all Italian SerDs were identified starting from the list available on the Health Ministry website and their HCPs invited to participate in the survey. With regard to hospital patients, they were reached through the Italian Association of hepatitis patients (EpaC), a representative of which has fully collaborated in the design and interpretation of the results of this study. Finally, HCPs invited patients of SerDs involved in the study. No fee was given to participants to complete the survey.

### Survey design

The survey was developed with the assistance of an independent third party (Calibra, Milan, Italy), providing methodological and statistical support, and then shared with the authors for discussion via several online meetings until a final agreement was reached. A cross-sectional survey was designed to collect information from clinicians and patients about participants’ characteristics and preferences for care pathways and possible treatments. The online survey was tested through pilot interviews to a small number of patients (n = 15) to assess comprehensibility of questions and the relevance of the pathway and treatment attributes and levels [18]. The final version of the surveys was based on the feedback received during the tests. The questionnaires were available online from July to November 2020.

The questionnaires dedicated to each study population were made available online through the Sawtooth Software platform which managed the randomization of attributes and levels of the DCE comparisons and the collection of responses.

The questionnaires were divided into four sections: a first section dedicated to collecting information on the socio-demographic characteristics of those who answered the questionnaire, a section dedicated to evaluating the characteristics of the care pathway through a series of questions to be answered ranking the possible answers in order of importance, two sections with DCE comparisons, the first dedicated to the evaluation of comparisons between possible care pathways for the positive HCV patient and the second dedicated to the evaluation of comparisons between treatment characteristics (Additional file 1: Fig. S1A, B). Results of the second part (analysis of ranking data and of the concordance between ranking and DCE data) are not the object of the present paper and will be presented in a next report.

Eight different discrete-choice experiments were developed: two domains (1. Care pathway, 2. Therapy), two settings (1. Hospital, 2. SerD), two stakeholders (1. Clinicians/HCPs, 2. Patients).

Attributes and levels were defined by a scientific board in four web-meetings, lasting each one about 2 h. The scientific board comprised two infectious disease specialists, three hepatologists, one SerD clinician, one patients’ representative, and one biostatistician. The set of questions was customized to each population (hospital clinicians, SerD HCPs, hospital patients, SerD users) according to abilities and interests, to maximize understanding of the questionnaire and information retrieval.

#### Care pathway

Eight attributes for hospital clinicians were chosen: Time-to-test, Time-to-taking-charge, Time-to-treat, Compliance-to-test, Compliance-to-taking-charge, Compliance-to-treatment, Diagnostic tests, Monitoring path and a ninth attribute was added for SerD HCPs: Care setting. These attributes were chosen to decompose the whole Meet–Test–Treat pathway, distinguishing three phases: from information to test, from test to diagnosis, from diagnosis to treatment. Each phase was further described by two domains: Time and Compliance. In addition, the profile of the possible pathways was enriched by an attribute about the diagnostic procedures and an attribute about the intensity of monitoring during therapy. The levels of time and compliance attributes were chosen in order to indicate an optimal (or suboptimal) level (e.g. a compliance of 90%), an unsatisfactory level (e.g. a compliance of 50%) and an intermediate level (e.g. a compliance of 70%). The levels of the other attributes were chosen by the scientific board to represent the typical diagnostic assessment and monitoring in Italy. Finally, the levels of attribute “Care setting” (specific for SerD HCPs) were “inside the service” and “outside the service”, to estimate the importance of testing and treating patients without the necessity to send them in external facilities.

Thinking to patients, the scientific board decided to reduce the dimension of profiles and thus to simplify choice tasks. Therefore, the three “time” attributes were collapsed in one, with levels approximately equal to the sum of the levels used for clinicians (the attribute Time Meet–Test–Treat was characterized by three levels: 2, 6 and 12 months). Compliance attributes were proposed identically to patients and clinicians, as well as the attributes “Monitoring path” and “Care setting” (only for SerDs). As regard to the attribute “Diagnostic tests”, the levels were a little bit simplified for hospital and SerD users. Table S1 (Additional file 1) reports the complete structure of Care pathway DCE (1.a, 1.b) and Therapy DCE (1.c, 1.d).

#### Treatment

Usually, DCE attributes of pharmacological therapy belong to three general categories: efficacy, side effects, modality of administration/monitoring. The scientific board decided to avoid the efficacy attribute since this is very high with currently available therapies and without a relevant variability. Modality of administration was indexed by “Number of pills per day”, “Duration”, “Concurrent use of other drugs”. These attributes were proposed both to clinicians and patients and with identical levels. For clinicians, modality of administration was further explored by four binary attributes: “Necessity of genotyping”, “Schedule”, “Administrable regardless of hepatic status”, “Administrable regardless of extra-hepatic diseases”. In addition, “Side effects”, with three levels of probability completed the features of therapies. For patients, besides the attributes shared with clinicians, modality of administration was explored by “Taking drugs” (with respect to meals). As regard to side effects, the scientific board decided to detail the typical side effects of anti-HCV therapies (diarrhea, headache, nausea) in order to evaluate their relative importance as choice drivers.

The random choice tasks were generated by Choice-Based Conjoint (CBC) method, available with Lighthouse Studio 9 (Sawtooth Software, Version 9.8.1). With regard to Care Pathway, eight random tasks were generated for clinicians and SerD HCPs, according to the “balanced overlap” method and, on the basis of indications obtained by testing the survey, a partial-profile design was applied, showing six (out of eight) attributes to hospital clinicians and seven (out of nine) to SerD HCPs. Tasks consisted in choosing obligatorily one of two profiles (combinations). For hospital patients and SerD users, six random tasks were generated, requiring to choose one of two profiles characterized by respectively four (out of six) and five (out of seven) attributes, thanks to a partial profile design.

With regard to Therapy, eight random tasks were generated for clinicians and SerD HCPs. In this case, a partial profile design was not applied, since in the pilot survey respondents did not show any problem in managing profiles characterized by eight attributes. Similarly, six random tasks were generated for hospital and SerD patients, again without applying partial profile design, thus showing profiles with all seven attributes identified to characterize therapies.

For each survey, sample size was defined by applying the “Test Design” tools of Sawtooth software, which use simulated data and compute logit efficiency, resulting in an estimate of standard errors for each attribute level. The general guideline is to achieve standard errors (SE) below 0.05. Considering also feasibility, based on factors such as number of specialists and of SerD staff potentially available, we slightly relaxed this requirement and planned surveys with 200 hospital clinicians (maximal estimated SE = 0.056), 200 SerD HCPs (maximal estimated SE = 0.057), 350 hospital patients (maximal estimated SE = 0.073) and 200 SerD users (maximal estimated SE = 0.094).

### Data analysis

DCE data were analyzed with the multinomial logit model, which pools respondent data in a single aggregate model, allowing to estimate effects, also called “part-worth utilities” in conjoint analysis. Such utilities refer to degrees of worth or preference for a feature. Within each attribute, the effects sum to zero. That is because one level for each attribute is omitted in doing the estimation, and then supply a value afterward for the missing level that is equal to the negative of the sum of the others. Thanks to this “effect coding” positive (negative) part-worth utility for a given level can be interpreted as an index of its desirability (undesirability) and the higher (the lower) the utility, the more (the less) desirable the attribute level.

The standard errors for each effect-used to compute 95% confidence intervals—are taken from the matrix of covariances among estimates obtained by inverting a sum of squares and cross-products matrix.

## Results

Out of 415 invited clinical centers, 190 responded and out of 955 invited SerD, 142 responded. The survey was completely answered by 190 hospital clinicians (73% of those who have started to fill in the questionnaire), 142 SerD HCPs (76%), 372 hospital patients (28%), and 131 SerD patients (61%). With respect to the planned sample size, the target was almost reached for hospital clinicians (190/200) and fully reached for hospital patients (372/350). However, we had a harder time recruiting all planned SerD HCPs (142/200) and patients (131/200). The large majority of respondents who did not complete the survey stopped at the very first pages of the online survey (title page or presentation or first question) and no information (age, sex, education, job, medical history, site of residence) is available to compare completers and non-completers. Respondents were located throughout the Italian territory and only two regions, accounting for a limited population (Molise and Valle d’Aosta), were not represented.

Characteristics of respondents are shown in Table 1.

**Table 1:** Characteristics of subjects who participated in the study: hospital clinicians, SerD HCPs, and hospital patients and SerD patients

			Hospital clinicians	SerD
*HCPs*				
Survey completers	n (%)		190 (73%)	142 (76%)
Age	Years, mean (SD)		54.1 (9.6)	58.0 (7.2)
Sex	Female, n (%)		79 (42%)	73 (51%)
Specialty	Hepatology: n (%)		23 (12%)	3 (2%)
	Gastroenterology: n (%)		51 (27%)	7 (5%)
	Infectious diseases: n (%)		91 (48%)	13 (9%)
	Internal Medicine: n (%)		43 (23%)	14 (10%)
	Other: n (%) More frequent: n (%)		15 (8%) Gerontology: 3 (20%)	107 (75%) Psychiatry: 31 (29%)
Experience in HCV	< 3 years, n (%)		4 (2%)	3 (2%)
	3–5 years, n (%)		4 (2%)	4 (3%)
	6–10 years, n (%)		16 (8%)	5 (4%)
	> 10 years, n (%)		166 (87%)	130 (91%)
*Patients*				
Survey completers	n (%)		372 (28%)	131 (61%)
Age (years)	Mean (SD)		61.5 (9.6)	47.4 (10.4)
Sex	Female: n (%)		149 (40%)	26 (20%)
Level of education	Primary school: n (%)		74 (20%)	84 (64%)
	High school: n (%)		182 (49%)	36 (28%)
	Master’s or bachelor degree: n (%)		115 (31%)	11 (8%)
Current condition	Sick patient: n (%)		33 (9%)	
	In treatment: n		8 (2%)	
	Cured patient: n (%)		339 (91%)	
	treatment duration, months: median (range)		3 (1–12)	
Substance addiction type	Alcohol addicted: n (%)			28 (21%)
	Drug addicted: n (%)			103 (79%)
Hepatitis C	Diagnosed: n (%)		372 (100%)	103 (82%)

Hospital clinicians had different specializations (some multiple specializations), all dedicated to medical care and treatment of physical diseases. SerD HCPs included non-physicians and medical doctors specialized in psychiatry. Although SerD HCPs included non-physicians, all respondents had clinical experience with HCV patients (i.e., psychological support to infected subjects). Most hospital patients had a past experience of HCV infection and were cured at the time of the survey. Most SerD patients had been diagnosed with HCV before the survey.

## Preferences for Meet–Test–Treat approach: care pathway

### Hospital clinicians

**Fig. 1:**
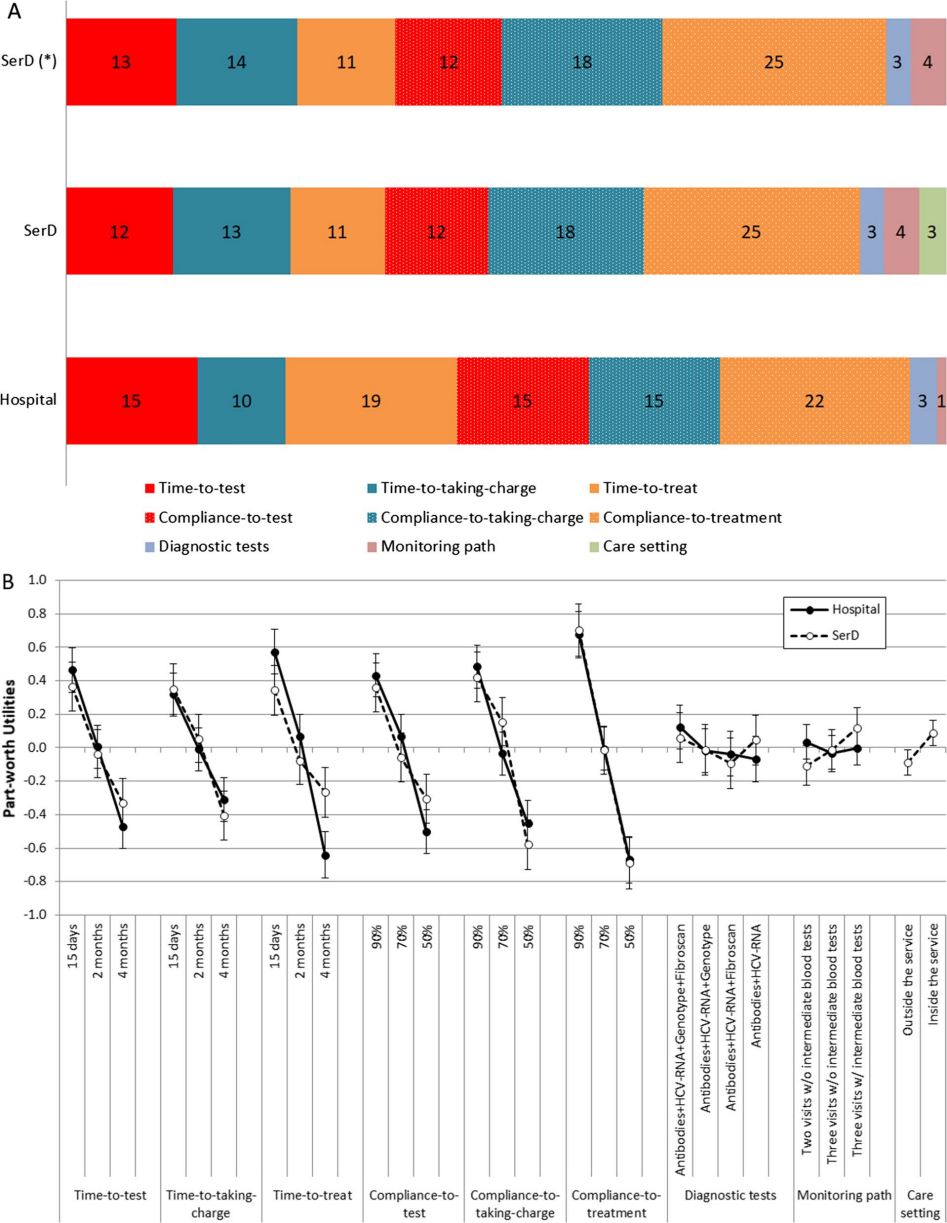
**A** Relative importance (%) of investigated attributes for the choice of a Meet–Test–Treat pathway for HCV management ((*) Percentages recalculated after excluding the attribute Care setting), and **B** preference weight for levels of investigated attributes for the choice of the Meet–Test–Treat pathway, according to hospital clinicians and SerD HCPs

On the basis of answers from hospital clinicians (Fig. 1A), the most important attribute of a suitable diagnostic–therapeutic pathway for detecting and efficiently treating HCV infection was a high “compliance to treatment” (relative importance [RI] 22%), which is defined by the proportion of subjects taking the prescribed therapy.

The second attribute of a suitable pathway was “time to treat” (RI 19%), which indicates the time from detection of HCV infection to initiation of therapy. Three attributes were deemed to have comparable importance (RI 15%): “compliance to test” (informed subjects/tested subjects), “time to test” (time from information about the possibility to be tested for HCV to performance of the test), and “compliance to taking charge” (patients who continue the diagnostic process/patients after testing positive for HCV). Among “compliance” and “time” attributes, less relevance was given to “time to taking charge” (time from a positive HCV test to further diagnostic analysis), which accounts for 10% of total importance. Lower importance (< 4%) was given to the other factors: “diagnostic tests” (expressing the level of diagnostic accuracy), and “monitoring pathway” (determined by the frequency of visits and tests in the pathway) (Fig. 1A).

The preference weights for levels of attributes were also studied (Fig. 1B, closed circles). For hospital clinicians, a high “adherence to treatment”, defined as a treatment administered to at least 90% of prescribed patients, was the preferred feature of a Meet–Test–Treat pathway (preference weight =  + 0.68; 95% CI + 0.54 to 0.81); preference weight was very high as expressed by the large point score difference between 90 and 50% adherence to treatment. Starting the treatment within 15 days from prescription, considered a short “time to treatment”, had the second level of importance (preference weight =  + 0.57; 95% CI + 0.44 to 0.71). Accordingly, a delay of 4 months was not acceptable, with a preference weight = − 0.64; 95% CI − 0.50 to − 0.78. A slightly lower but relevant preference weight was given to a good compliance to test, a short time to test, a high compliance to pursue the diagnostic procedure (“taking charge”), and a short time to in-depth diagnostic procedure (“time to taking charge”).

Diagnostic tests and types of monitoring were not very relevant, with all levels equally considered, as shown by overlapping confidence intervals for both attributes. Only a weak although nonsignificant preference for complete diagnostic assessment (antibodies + HCV-RNA + genotype + Fibroscan) was observed, but this attribute's importance in choosing the optimal Meet–Test–Treat pathway can be considered negligible with respect to adherence and timing features.

### SerD HCPs

The questionnaire for SerD HCPs included an attribute that was not proposed to hospital clinicians: the possibility of being tested, assessed and treated for HCV either inside or outside the habitual SerD (“care setting”).

The estimation of relative importance of attributes by SerDs HCPs was similar but not identical to the one by hospital HCPs (Fig. 1A). The most important attributes of a suitable pathway were “adherence to treatment” (RI 25%), and compliance to the diagnostic assessment (RI 18%). Similar relevance had the time to diagnosis (RI 13%), time to test (RI 13%), compliance to test (RI 12%), and time to treatment (RI 11%). The monitoring pattern, high accuracy of diagnostic analysis, and the care setting were considered less important. Even if the inclusion of an additional attribute in one questionnaire does not strictly compare data from a hospital and SerD HCPs, in Fig. 1 the RI was recalculated, excluding the last attribute for SerD HCPs (SerD*). Comparing the bars “hospital” and “SerD(*)”, the sum of the three adherence attributes reaches 51.4% for hospital and 55.7% for SerD HCPs, while the sum of three-time attributes is larger for hospital clinicians (44.4%) than for SerD HCPs (37.4%). More in detail, this difference could be mainly explained by the reduction of the relative importance for time to treat from 19% for hospital clinicians to 11% for SerD HCPs, who considered more important the time to taking charge (14%).

For SerD HCPs, the analysis of preference weights for levels of attributes (Fig. 1B, open circles) shows that although the care setting was not deemed important in comparison with other attributes, a significant preference for “inside the service” versus “outside the service” was present (preference weight =  + 0.09; 95% CI + 0.01 to 0.16).

Preference weights for levels of attributes by SerD HCPs and hospital clinicians were similar (Fig. 1B, open circles). A longer time to treat (the level 4 months) was less unacceptable for SerD HCPs (preference weight = − 0.27; 95% CI − 0.41 to − 0.12) and closer to the level 2 months (preference weight = − 0.08; 95% CI − 0.22 to 0.06), in comparison with hospital clinicians. The RI of diagnostic tests or monitoring path was low (3% and 4%, respectively) and the corresponding lines were virtually flat.

### Hospital patients

**Fig. 2:**
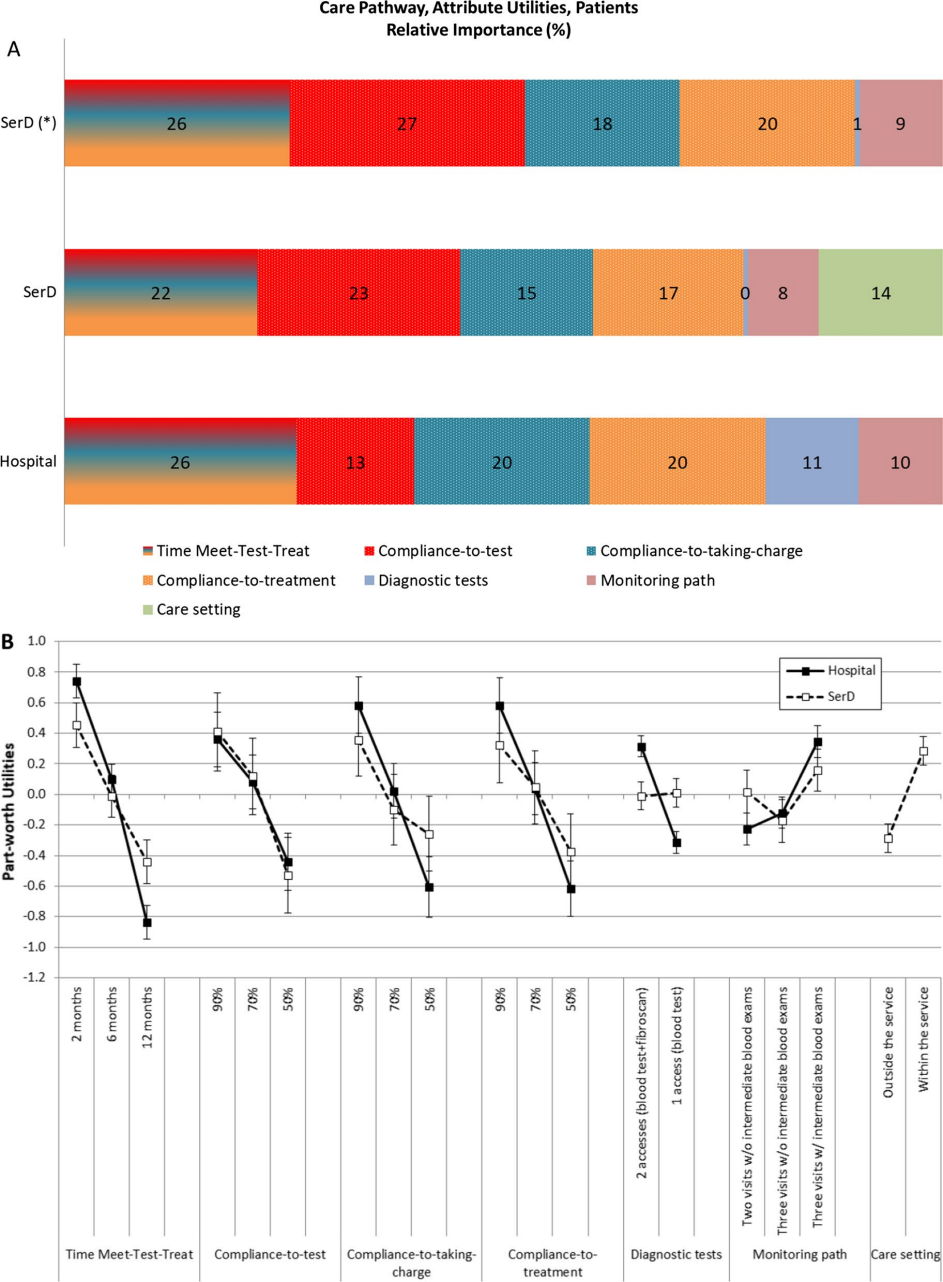
**A** Relative importance (%) of investigated attributes for the choice of a Meet–Test–Treat pathway for HCV management ((*) Percentages recalculated after excluding the attribute Care setting), and **B** Preference weight for levels of investigated attributes for the choice of the Meet–Test–Treat pathway, according to hospital and SerD patients

Among subjects currently (9%) or previously (91%) affected by HCV infection, the evaluation of the RI of attributes (Fig. 2A) showed that the duration of the whole diagnostic–therapeutic pathway (from meeting to testing and treating) was the most important attribute (RI 26%). It should be borne in mind that, as described in ‘Methods’, the attribute “time”, fractioned in three parts in the questionnaire for clinicians, was presented as a single attribute for patients. Compliance with the diagnostic assessment and adherence to treatment had comparable importance (RI 20%). The other attributes were less relevant: compliance to test (RI 13%), the relative importance of diagnostic (RI 11%) and importance of monitoring path (RI 10%).

Looking at preference weights for levels of attributes (Fig. 2B, closed squares), the higher weight was given to a 2-month delay between the proposal of HCV test and initiation of treatment, with a strongly negative weight (disapproval) for a 12-month delay, and with significant differences among levels of this time attribute. Comparable results were obtained for compliance to diagnostic assessment and adherence to treatment, which had high point scores for the 90% level and significantly lower point scores for 70% and 50% levels. Conversely, for compliance to test, the difference between 90 and 70% levels was not significant and smaller than the difference between 70 and 50% levels. The preference weight for a pathway characterized by 90% of patients accepting to be tested was about 0.2 points smaller than the preference weight for a pathway with 90% of patients accepting to follow the diagnostic procedure and be treated in case of a confirmed diagnosis of HCV. A high preference was observed for examination with Fibroscan in addition to blood test versus blood tests only, even if two hospital accesses would be necessary instead of one. Likewise, these subjects reported a high preference for strict monitoring, including three visits during the treatment plus intermediate blood tests, versus less stringent monitoring.

### SerD patients

The questionnaire for SerD patients included an attribute that was not proposed to hospital patients: the possibility of being tested, assessed and treated for HCV either inside or outside the habitual SerD (“care setting”).

For addicted subjects who were followed-up by SerD, the most important attribute of a suitable diagnostic, therapeutic pathway for HCV was compliance to test (RI 23%) (Fig. 2A). The duration of the whole Meet–Test–Treat pathway was deemed to be almost equally important (RI 22%), and adherence slightly less important (RI 17%). Decreasing importance was given to compliance to diagnostic assessment (RI 15%), receiving treatment for HCV within the SerD “care setting” (RI 14%), monitoring pathway (RI 8%), and the number of accesses for diagnostic procedures (RI 0.48%).

The analysis of preference weights for levels of attributes in this group (Fig. 2B, open squares) showed that the possibility to complete the whole Meet–Test–Treat pathway in a shorter time, compliance to diagnostic assessment, and adherence to treatment were appreciated although not so important as for hospital patients. A high compliance to test had a high level of importance, as for the hospital patients. These patients showed no preference for either type of diagnostic procedure, being indifferent to the number of accesses and the use of Fibroscan. Accordingly, these subjects seemed indifferent to the stringency of monitoring for HCV. Finally, SerD patients showed a significant preference for being cared for HCV within the SerD versus being cared for in other centers (preference weight =  + 0.29; 95% CI + 0.19 to 0.38).

## Preferences for Meet–Test–Treat approach: treatments

### Hospital clinicians

**Fig. 3:**
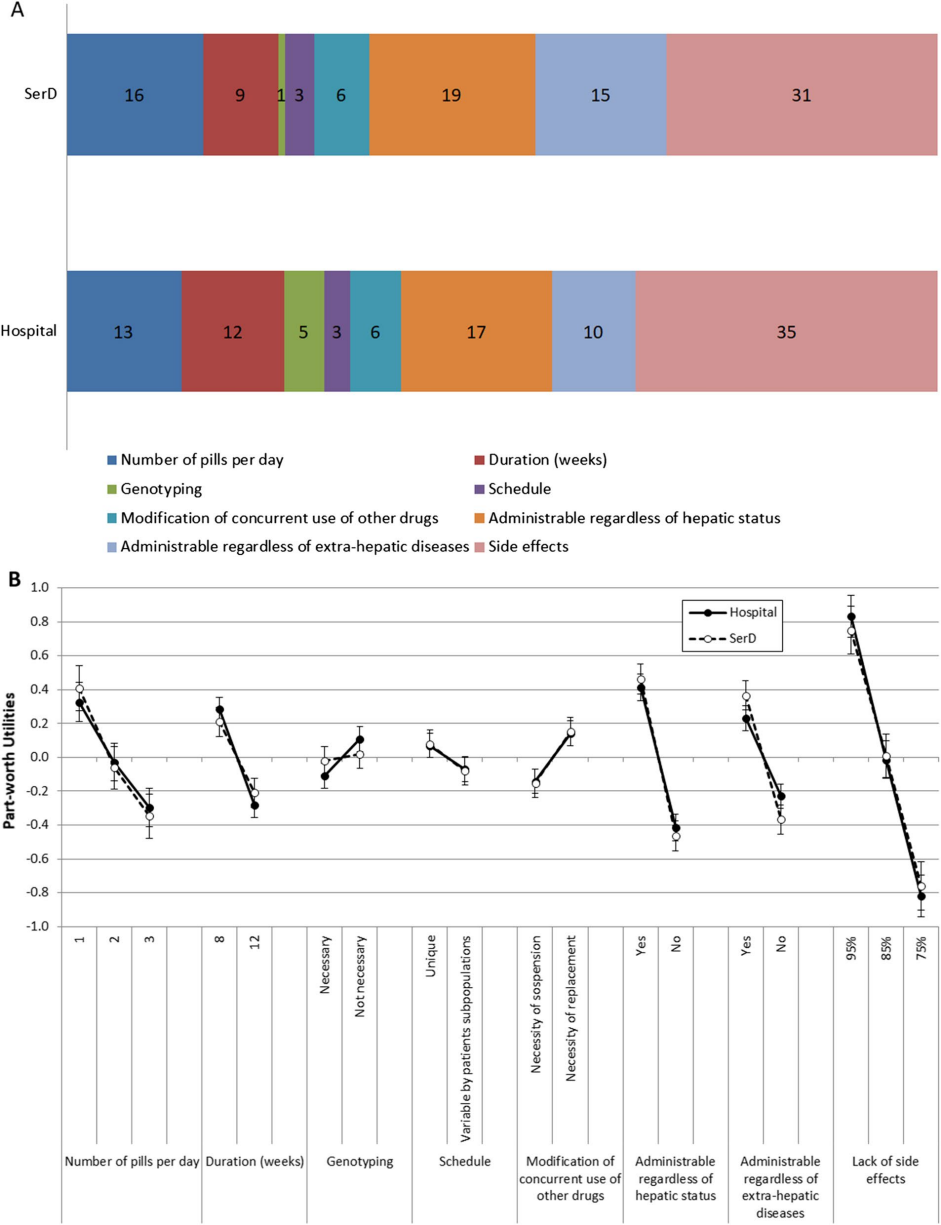
**A** Relative importance (%) of investigated attributes for the choice of treatment for HCV, and **B** Preference weight for levels of investigated attributes for the choice of treatment for HCV, according to hospital clinicians and SerD HCPs

The relative importance of treatment attributes for hospital clinicians is shown in Fig. 3A. They choose a treatment for HCV mainly based on the tolerability profile (RI 35%). They also give importance to the possibility of administering therapy regardless of hepatic status (RI 17%) and to the number of pills per day (RI 13%). The importance of therapy duration (RI 12%) is slightly lower. The other attributes are progressively less important (below 6% RI).

Exploring the preference weights of each level of attributes, good tolerability is confirmed as the main driver for choice (Fig. 3B, closed circles). The preference weight for a 95% probability of no side effects was =  + 0.83; 95% CI + 0.71 to 1.08, for possibility to treat regardless of hepatic status =  + 0.41; 95% CI + 0.16 to 0.38, and for the administration with 1 pill per day =  + 0.33; 95% CI + 0.21 to 0.56. This score was slightly lower for a shorter therapy duration (+ 0.28; 95% CI + 0.07 to 0.21) and possible use regardless of extra-hepatic diseases (+ 0.23; 95% CI + 0.16 to 0.38).

### SerD HCPs

As evident in Fig. 3A, B SerD HCPs and hospital clinicians essentially agreed about the weights of therapy choice drivers. Only two peculiarities are worthy of being reported. The small importance hospital clinicians attributed to the necessity of genotyping before deciding the therapy vanished completely for SerD HCPs. The preference weight for possibility to treat regardless of extra-hepatic diseases increased from + 0.23 for hospital clinicians + 0.37 (95% CI + 0.28 to 0.54) for SerD HCPs.

### Hospital patients

**Fig. 4:**
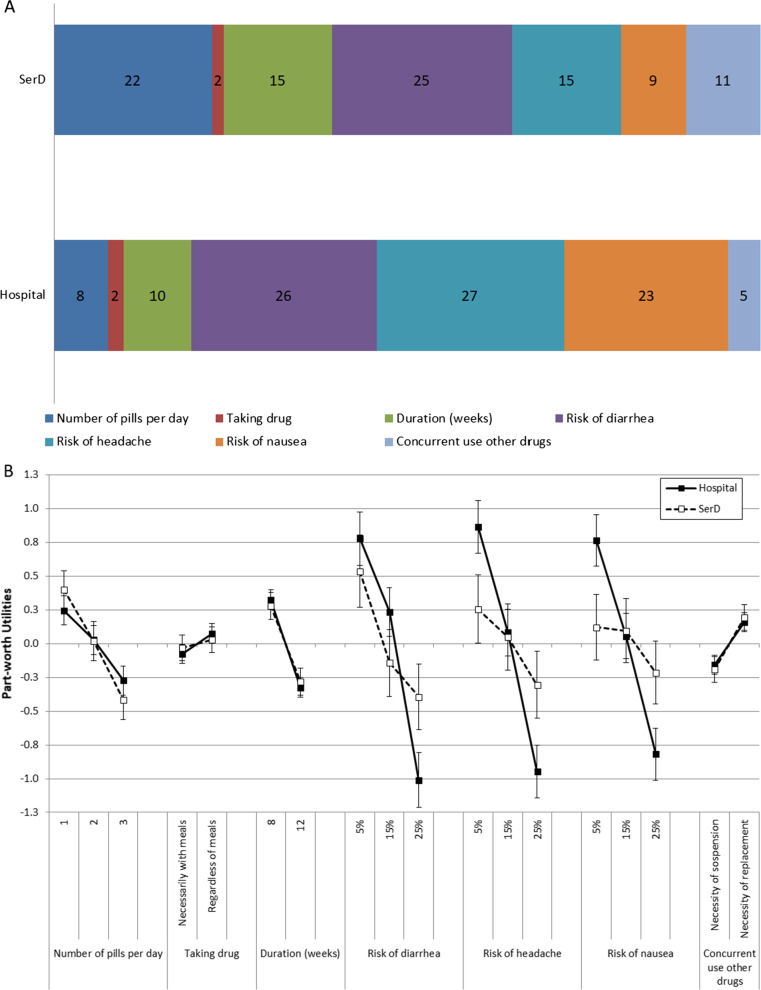
**A** Relative importance (%) of investigated attributes for the choice of treatment for HCV, and **B** Preference weight for levels of investigated attributes for the choice of treatment for HCV, according to hospital and SerD patients

In agreement with hospital clinicians, for hospital patients, the preferred characteristic of treatment for HCV was the low risk of side effects (Fig. 4A). In the patients’ questionnaire, specific typical side effects were presented: diarrhea, headache and nausea. Their RI was similar (headache = 27%, diarrhea = 26%, nausea = 23%). Among the other attributes, duration (RI 10%) and number of pills (RI 8%) were almost similarly important. “Concurrent use of other drugs” (RI 5%) and “prescription of a new drug” (RI 2%) was less relevant.

The profile of preference weights according to levels of attributes (Fig. 4B, closed circles) confirmed the high relevance of tolerability, as shown by the large point score difference between the preferred and the undesirable levels for risk of diarrhea, risk of headache, and risk of nausea. 8 weeks were preferred to 12 weeks, one pill to three pills and replacement therapy to suspension.

### SerD patients

While hospital clinicians and SerD HCPs reported similar preferences, SerD patients valued treatment attributes differently with respect to hospital patients. The RI was high and similar only for the risk of diarrhea (26% for hospital and 25% for SerD patients). The risk of headache was less important for SerD patients than for hospital patients (15% RI vs 27% RI, respectively); similarly, the risk of nausea (9% RI for SerD, and 23% RI for hospital patients) (Fig. 4A). On the contrary, the number of pills per day had 22% RI for SerD patients and was even more important than for hospital patients (8% RI). For SerD patients, side effects accounted for a small percentage of total importance, and the remaining percentage was mainly absorbed by number of pills.

At the analysis of weights for attribute levels (Fig. 4B, open circles), all side effects attributes showed a lower difference between each attribute’s preferred and undesirable levels compared to hospital patients. Instead, SerD patients appreciated the low number of pills per day even more than hospital patients. It is also confirmed that good tolerability seemed slightly less important for SerD patients than hospital patients.

## Discussion

This survey investigated the attitude of four different populations involved with HCV management toward a Meet–Test–Treat strategy aiming to maximize the identification of subjects at risk of HCV infection and increase compliance to diagnostic tests and treatment of infected cases. This approach has the objective of eradicating HCV and has been proposed since the availability of effective and safe pharmacological treatment for HCV.

The respondents were representative of most of the main actors involved in the management approach, in the Italian National Health System, including HCPs and lay people from two different settings: hospitals where HCV is cured, and SerD, where subjects find a persistent contact with the healthcare system. Our DCE survey addressing eight different scenarios obtained a thorough picture of the real-life expectations for a program toward HCV eradication. Almost all previous DCE surveys about either HCV or HBV evaluated preferences for one issue alone (treatment, vaccination, primary care service), by a single perspective perspective [19–23]. Pacou et al. [24] carried out a DCE in the UK to compare patients’ and physicians’ perspectives about treatment for HCV, showing that efficacy was the preferred driver of choice, with greater importance for physicians than patients.

Time to treatment (the time from first contact to initiation of treatment) is deemed important to the strategy’s success, by all actors, with little differences. A short time to treatment might be required for many reasons: to be cured as soon as possible, to reduce the interference of tests and treatments with working activities, to reduce the risk of withdrawal, to reduce costs, to facilitate the organization of care services [25, 26]. Indeed, the time to treatment is less important for the SerD HCPs than for hospital clinicians, probably due to their different patients: the hospital patient aims at being cured and followed-up to avoid complications, but expects the HCV pathway to have an end, while the SerD patient will attend the center independently of the HCV pathway for a long-term period.

Patients affected with HCV have a very different relationship with the professionals of the health system depending on whether they are followed at the hospital or st the SerD, and as a consequence, HCPs working in these two settings have special strategies to deal with each type of patient. Using a quantitative methodology, a complex eight DCE study assessed differences and similarities of preferences among the four groups of actors. The authors choose to explore two main features of a possible program aimed at eradicating HCV: the clinical pathway from access to testing up to access to treatment and treatment itself.

Looking forward to the organization of the care system, identification of the most effective location for the interventions is of extreme relevance. Based on our results, different styles of healthcare should be offered to the general population and the key population, here represented by SerD patients. While hospitals and medical units are the obvious references for many infected patients, the location suitable to meet at-risk subjects, who need to be guided toward testing, is not so obviously identified. Despite the relative importance given to “care setting”, SerD patients gave an evident preference for being cared within the addiction centers, which are familiar to them, and which they regularly attend. This is also important for SerD HCPs who are a strong reference for their patients; hospital clinicians were less aware of the importance of the patient-HCP relationship in this process. This preference seems to require attention from the institutions, as it may impact on the compliance and as a whole on the possibility to contact and treat the main infection source. To answer this need, healthcare providers should develop new diagnostic and therapeutic pathways taking advantage of new tools, such as telemedicine to improve patients’ linkage to therapy. A care pathway for HCV within addiction centers will only be available if professional skills are improved and specialized medical competence is provided. It is possible that infectious disease specialists from a joint HCV center may regularly move to SerDs for visits of addicted patients, preventing patient removal from his/her habitual environment. These results may confirm findings by Radley et al. [21], taking into account the different organizations of the health system in Italy and UK; these authors showed that addicted subjects on opioid substitution therapy prefer testing at their own pharmacy, suggesting that good relationships between patients and test providers may improve compliance to testing. In Italy, a program of dedicated transportations and cure has been proposed for patients with substance use disorder and HCV who live in a geographic area not very well served by public transport; this area is served by 15 SerDs and only one hepatology unit, so that deficient public transportation leads to a logistical barrier to access needed services (ClinicalTrials.gov Identifier: NCT03923595).

The attitude of hospital patients highlighted their preference for a pathway involving the use of transient elastography; they expect to be closely analyzed, diagnosed and finally cured; this result may also be due to the majority of these patients having been treated before highly effective and safe regimens were available, allowing simple pre-treatment evaluations; on the contrary, SerD patients are less aware to the process, and to the accuracy of evaluation.

On the basis of both hospital clinicians and SerD HCPs, tolerability is shown as the most important feature of a suitable treatment. Absence of side effects, possibility to administer regardless of other liver diseases, and of a liver condition were considered extremely important for a therapy to be proposed to patients. In addition, the administration of one pill per day was also appreciated by clinicians and SerD HCPs. Hospital clinicians answered that no necessity of genotyping was also a favorable attribute, while this characteristic was irrelevant for SerD clinicians.

In the opinion of hospital patients, a suitable treatment for HCV must have a very low risk of headache, diarrhea, and nausea; all the other characteristics are relatively less important, including the number of pills per day and modification of concurrent use of other drugs. It may be speculated that these patients desire a tolerable treatment to not interfere with their daily activities.

The SerD patients showed a different attitude toward treatment characteristics; they were less interested in the absence of adverse events than hospital patients, and the importance of tolerability was not higher than the importance of the number of pills per day. Indeed, opioid use or substitution is likely to reduce the risk for such side effects.

A previous DCE survey by Welzel et al. [19] investigated patients' preferences in the USA and Europe, about treatment for HCV. Patients significantly preferred a DAA regimen with a higher cure rate, shorter treatment duration, lower risks of diarrhea, headache and nausea (all p < 0.001), reduced need for office visits when on treatment (p = 0.044), and without requiring dose reduction (p = 0.032). This study may be compared only with the section of our survey directed to hospital patients and evaluating treatment preferences. The results mainly agree with our findings, although we did not evaluate the importance of a high cure rate, which is nowadays a shared feature of DAAs.

In conclusion, this survey showed the preferences of clinicians and patients concerning attributes related to socio-demographic characteristics, importance of the care pathway characteristics, comparisons between possible care pathways for the positive HCV patient and comparisons between treatment characteristics. Policymakers and all others involved should take into account these preferences when planning a Meet–Test–Treat strategy. This study takes into account the needs and preferences of most of the actors in the process, in order to optimize it. As a whole, peculiar needs of patients followed-up by SerDs were identified, and these subjects should be met in healthcare structures familiar to them. In the joint perspective of HCPs and of lay people, the diagnostic process should be rapid and thorough, and treatment should be tolerable and accessible, while treatment efficacy is taken for granted.

## Supplementary Information

**Additional file 1.** List S3.

## Data Availability

Data are available to be shared with other researchers. The datasets area available from the corresponding author on reasonable request.

## Abbreviations

DAA: Direct-acting antiviral
DCE: Discrete choice experiment
HCP: Healthcare provider
HCV: Hepatitis C virus
PWID: People who inject drugs
SerD: Public drug addiction service
WHO: World Health Organization
