# Supplementary material for: Meet–Test–Treat for HCV management: patients’ and clinicians’ preferences in hospital and drug addiction services in Italy
Source: BMC Infect Dis. 2022 Jan 4;22:3. doi: 10.1186/s12879-021-06983-y (PMC8725306; doi:10.1186/s12879-021-06983-y)
Supplement: Supplementary file 1 — Additional file 1. List S3. [file 12879_2021_6983_MOESM1_ESM.docx]

Additional Material

1. **Attributes and Levels**

**Table S1.a - Care Pathway attributes and levels for hospital clinicians and SerD HCPs.**

| **Attribute** | **Level** |
| --- | --- |
| Time-to-test | 15 days |
|  | 2 months |
|  | 4 months |
| Time-to-taking-charge | 15 days |
|  | 2 months |
|  | 4 months |
| Time-to-treat | 15 days |
|  | 2 months |
|  | 4 months |
| Compliance-to-test | 90% |
|  | 70% |
|  | 50% |
| Compliance-to-taking-charge | 90% |
|  | 70% |
|  | 50% |
| Compliance-to-treatment | 90% |
|  | 70% |
|  | 50% |
| Diagnostic tests | Antibodies + HCV-RNA + Genotype + Fibroscan |
|  | Antibodies + HCV-RNA + Genotype |
|  | Antibodies + HCV-RNA + Fibroscan |
|  | Antibodies + HCV-RNA |
| Monitoring path | Two visits w/o intermediate blood tests |
|  | Three visits w/o intermediate blood tests |
|  | Three visits w/ intermediate blood tests |
| Care setting* | Outside the service |
|  | Inside the service |

*This attribute was considered only for SerD HCPs

**Table S1.b - Care Pathway attributes and levels for hospital patients and SerD users.**

| **Attribute** | **Level** |
| --- | --- |
| Time Meet-Test-Treat | 2 months |
|  | 6 months |
|  | 12 months |
| Compliance-to-test | 90% |
|  | 70% |
|  | 50% |
| Compliance-to-taking-charge | 90% |
|  | 70% |
|  | 50% |
| Compliance-to-treatment | 90% |
|  | 70% |
|  | 50% |
| Diagnostic tests | 2 accesses (blood test + Fibroscan) |
|  | 1 access (blood test) |
| Monitoring path | Two visits w/o intermediate blood exams |
|  | Three visits w/o intermediate blood exams |
|  | Three visits w/ intermediate blood exams |
| Care setting* | Outside the service |
|  | Within the service |

*This attribute was considered only for SerD users

**Table S1.c - Therapy attributes and levels for hospital clinicians and SerD HCPs.**

| **Attribute** | **Level** |
| --- | --- |
| Number of pills per day | 1 |
|  | 2 |
|  | 3 |
| Duration (weeks) | 8 |
|  | 12 |
| Genotyping | Necessary |
|  | Not necessary |
| Schedule | Unique |
|  | Variable by patient subpopulations |
| Concurrent use of other drugs | Necessity of suspension |
|  | Necessity of replacement |
| Administrable regardless of hepatic status | Yes |
|  | No |
| Administrable regardless of extra-hepatic diseases | Yes |
|  | No |
| Lack of side effects | 95% |
|  | 85% |
|  | 75% |

**Table S1.d - Therapy attributes and levels for hospital patients and SerD users.**

| **Attribute** | **Level** |
| --- | --- |
| Number of pills per day | 1 |
|  | 2 |
|  | 3 |
| Taking drug | Necessarily with meals |
|  | Regardless of meals |
| Duration (weeks) | 8 |
|  | 12 |
| Risk of diarrhea | 5% |
|  | 15% |
|  | 25% |
| Risk of headache | 5% |
|  | 15% |
|  | 25% |
| Risk of nausea | 5% |
|  | 15% |
|  | 25% |
| Concurrent use other drugs | Necessity of suspension |
|  | Necessity of replacement |

1. **Exemplary DCE task cards**

**Figure S1 -** Exemplary DCE task cards: A) card proposed to SerD HealthCare Providers to evaluate attributes of care pathway. B) card proposed to hospital patients to evaluate attributes of therapy.

A

B

1. **Hospital clinicians and SerD HCPs who participated in the study**

**Hospital Clinicians**

ABRUZZO - Nerio Iapadre, Ospedale Regionale San Salvatore, Aquila - Malfisa Di Carlo, Claudio Ucciferri, Francesca Vignale, Ospedale SS. Annunziata, Chieti - Valeria Pace Palitti, Presidio Ospedaliero Penne, Penne (PE) - Pierluigi Cacciatore, Giustino Parruti, Ospedale S. Spirito, Pescara - Pierluigi Tarquini, Ospedale Mazzini, Teramo.

BASILICATA - Giacinto Casciano, Ospedale Madonna delle Grazie, Matera - Vito Carretta, Ospedale di Venosa, Venosa (PZ).

CALABRIA - Francesco Quintieri, AO Pugliese Ciaccio, Catanzaro - Benedetto Caroleo, Carlo Torti, AOU Mater Domini Policlinico Campus S. Venuta Germaneto, Catanzaro - Ileana Luppino, Mario Verta, Ospedale Annunziata, Cosenza - Maurizio Pino, ASP Magna Grecia Crotone - PO San Giovanni di Dio, Crotone - Giuseppina Berardelli, Lorenzo Antonio Surace, ASP CZ - PO Lamezia Terme, Lamezia Terme (CZ) - Giuseppe Foti, Alfredo Kunkar, PO Ospedali Riuniti, Reggio Calabria.

CAMPANIA - Addolorata Masiello, Ospedale San Giuseppe Moscati, Avellino - Angelo Salomone Megna, Ospedale G. Rummo, Benevento - Vincenzo Messina, Ospedale Sant’Anna e San Sebastiano, Caserta - Daniela Caterina Amoruso, Maria Rosaria Barbella, Laura Staiano, PO Gragnano, Gragnano (NA) - Filomena Morisco, AOU Federico II Napoli - Giovan Giuseppe di Costanzo, Ospedale Antonio Cardarelli, Napoli - Vincenzo Narciso, Ospedale Ascalesi, Napoli - Andrea Fontanella, Luca Fontanella, Michele Imparato, Ospedale Buon Consiglio, Napoli - Amedeo Cecere, Ospedale Evangelico Villa Betania, ASL NA 1, Napoli - Vincenzo Iovinella, Ospedale San Paolo, Napoli - Carmela Loguercio, II Università degli Studi di Napoli (SUN), Policlinico, Napoli.

EMILIA ROMAGNA - Fabio Levantesi, Ospedale di Bentivoglio, Bentivoglio (BO) - Paolo Caraceni, Maria Cristina Morelli, Ilaria Serio, Gabriella Verucchi, Pierluigi Viale, AOU Policlinico S. Orsola-Malpighi, Bologna - Arianna Lanzi, Federica Mirici Cappa, Ospedale di Faenza, Faenza (RA) - Anastasio Grilli, Marco Libanore, Laura Sighinolfi, Loredana Simone, Arcispedale Sant’Anna, Ferrara - Cristina Davighi, Ospedale di Fidenza - San Secondo, Fidenza (PR) - Ilaria Manzi, Ospedale G.B. Morgagni - L. Pierantoni, Forlì - Andrea Lisotti, Ospedale S. Maria della Scaletta, Imola (BO) - Vanni Borghi, Nicola Cautero, Elena Corradini, AOU di Modena, Modena - Carmen Vandelli, Nuovo Ospedale Civile S. Agostino-Estense, Modena - Roberto Bertoni, Alessandra Orlandini, Universitaria di Parma, Parma - Alberto Faggi, Ospedale di Piacenza, Piacenza - Valentina Cesario, Marco Massari, Santa Maria Nuova di Reggio Emilia, Reggio Emilia - Antonio Boschini, Malattie Infettive di San Patrignano, Rimini.

FRIULI VENEZIA GIULIA - Giovanna Dettori, Elena Garlatti Costa, Ospedale S. Maria degli Angeli, Pordenone - Ivo Crosato, AOU Ospedali Riuniti - Ospedale Maggiore, Trieste - Davide Pecori, Ospedale Santa Maria della Misericordia, Udine - Debora Donnini, Giorgio Soardo, Pierluigi Toniutto, Ospedale Universitario Santa Maria della Misericordia, Udine.

LIGURIA - Valentina Bartolacci, PO Santa Maria della Misericordia, ASL 2, Albenga (SV) - Simona Marenco, Laura Ambra Nicolini, Antonio Di Biagio, IRCCS - AOU San Martino, Genova - Giovanni Cassola, Emanuele Pontali, Ente Ospedaliero Ospedali Galliera, Genova - Michele Guerra, Ospedale S. Andrea - ASL 5, La Spezia - Ilaria De Macina, Ospedale di Sanremo - ASL 1 Imperiese, Sanremo (IM) - Marco Anselmo, Pasqualina De Leo, Ospedale San Paolo, Savona.

LOMBARDIA - Isabella Carderi, AO Bolognini Seriate - Ospedale SS. Capitanio e Gerosa, Lovere (BG) - Gianni Gattuso, AO Carlo Poma, Mantova - Simona Bollani, AO Fatebenefratelli e Oftalmico, Milano - Guido Gubertini, Simona Landonio, Carlo Magni, Fosca Niero, AO Luigi Sacco, Milano - Chiara Mazzarelli, Giovanni Perricone, Massimo Puoti, Raffaella Viganò, Maria Vinci, AO Niguarda Ca’ Granda, Milano - Anna De Bona, Alessia Giorgini, Massimo Zuin, AO San Paolo, Milano - Maria Grazia Rumi, Ospedale Classificato San Giuseppe, Milano - Luca Aldrighetti, Massimo Memoli, Caterina Uberti-Foppa, Ospedale San Raffaele, Milano - Antonio Ciaccio, Alessandro Soria, AO San Gerardo, Monza - Raffaele Bruno, Fondazione IRCCS Policlinico San Matteo, Pavia - Barbara Omazzi, AO G. Salvini Garbagnate - Presidio Ospedaliero di Rho, Rho (MI) - Anna Francesca Panzeri, Ombretta Spinelli, AO Sant’Anna di Como, San Fermo Della Battaglia (CO).

MARCHE - Andrea Giacometti, Giuseppe Tarantino, Ospedali Riuniti Ancona, Ancona - Alessandro Chiodera, Ospedale Generale Provinciale, Macerata.

PIEMONTE - Valeria Barbon, Ospedale SS Antonio e Biagio, Alessandria - Carlo Smirne, AO Maggiore della Carità, Novara - Cosimo Colletta, ASL VCO - Ospedale Madonna del Popolo di Omegna, Omegna (VB) - Paolo Scivetti, Ospedale degli Infermi di Biella, Ponderano (BI) - Stefano Bonora, Giuseppe Cariti, Ospedale Amedeo di Savoia, Torino - Bianca Bianchi, ASL VC - Ospedale Sant’Andrea, Vercelli.

PUGLIA - Gianfranco Lauletta, Michele Milella, AOU Policlinico Consorziale Bari, Bari - Nicola Napoli, Gaetano Brindicci, Ospedale Policlinico di Bari, Bari - Giuseppe Cuccorese, Ospedale M. Dimiccoli, Barletta - Ruggero Losappio, Michele Mazzola, Carmen Rita Santoro, Ospedale Vittorio Emanuele, Bisceglie (BT) - Annamaria Longo, Ospedale Di Summa - Perrino, Brindisi - Nicola Minerva, Ospedale Caduti di tutte le guerre, Canosa (BT) - Raffaele Cozzolongo, IRCCS De Bellis, Castellana Grotte (BA) - Antonio Patrizio Termite, Ospedale di Castellaneta, Castellaneta (TA) - Sergio Cappello, ASL FG - Ospedale G. Tatarella, Cerignola (FG) - Simona Morella, Ospedali Riuniti, Foggia - Roberto Chiavaroli, Paolo Tundo, Ospedale Santa Caterina Novella, Galatina (LE) - Kwelusukila Loso, Fulvio Mastrandrea, PO Marianna Giannuzzi, Manduria (TA) - Salvatore Rizzo, ASL TA - PO Valle D’Itria - Stabilimento Ospedaliero di Martina Franca, Martina Franca (TA) - Emanuela Ciracì, ASL BR - Ospedale di Ostuni, Ostuni (BR) - Angelo Andriulli, Angelo Iacobellis, Anna Grazia Niro, Antonio Massimo Ippolito, Ospedale Casa Sollievo della Sofferenza, San Giovanni Rotondo (FG) - Egidio Visaggi, ASL BA - Ospedale M. Sarcone, Terlizzi (BA).

SARDEGNA - Paola Pisano, P.O. SS Trinità, Cagliari - Luchino Chessa, AOU Cagliari - Policlinico, Monserrato (CA) - Graziella Ogana, P.O. Giovanni Paolo II, Olbia - Sergio Babudieri, Ivana Maida, AOU di Sassari, Sassari - Salvatore Zaru, P.O. SS. Annunziata, Sassari.

SICILIA - Giuseppe Alaimo, ASP - PO S. Giovanni di Dio, Agrigento - Ignazio Scalisi, ASP Trapani - Ospedale Vittorio Emanuele II, Castelvetrano (TP) - Arturo Montineri, AOU Policlinico Vittorio Emanuele - Ospedale Ferrarotto Alessi, Catania - Gaetano Bertino, Evelise Frazzetto, AOU Policlinico Vittorio Emanuele - P.O. Gaspare Rodolico, Catania - Francesco Benanti, Mariarita Cannaò, Maurizio Russello, PO Garibaldi-Nesima, Catania - Antonio Di Giacomo, ASP Ragusa - Ospedale Regina Margherita di Comiso, Comiso (RG) - Luigi Guarneri, ASP Enna - PO Umberto I, Enna - Vincenzo Portelli, Francesca Savalli, ASP Trapani - Ospedale S. Antonio Abate, Erice (TP) - Irene Cacciola, Roberto Filomia, A.O.U. Policlinico G. Martino, Messina - Antonio Ficalora, A.R.N.A.S. Civico - Di Cristina - Benfratelli, Palermo - Giuseppe Malizia, AO Ospedali Riuniti Villa Sofia-Cervello - Ospedale Cervello, Palermo - Vincenza Calvaruso, Antonio Cascio, Vito Di Marco, Lydia Giannitrapani, Anna Licata AOU Paolo Giaccone, Palermo - Mario Mitra, PO Civico e Benfratelli, Palermo - Marco Di Stefano, ASP Siracusa - Ospedale Umberto I, Siracusa.

TOSCANA - Piera Pierotti, Ospedale Santa Maria Annunziata - ASL 10 Firenze, Bagno a Ripoli (FI) - Andrea Nuccorini, Giuseppe Parisi, Rodolfo Sacco, Ospedale Universitaria Pisana, Cisanello (PI) - Elisabetta Lorefice, Ospedale San Giuseppe, Empoli (FI) - Giuseppe Indolfi, AOU Meyer, Firenze - Dario Bartolozzi, Giampaolo Corti, Mirko Tarocchi, AOU Careggi, Firenze - Roberta Cinelli, Presidio Ospedaliero Livorno, Livorno - Alessandro Nerli, Ospedale Civile Misericordia e Dolce, Prato - Barbara Rossetti, Giacomo Zanelli, Daniele Marri, AOU Senese - Policlinico Santa Maria alle Scotte, Siena.

TRENTINO ALTO ADIGE - Lorenza Guella, Giovanni De Pretis, Ospedale S. Chiara, Trento.

UMBRIA - Fabio Chistolini, Federica Sannella, Poliambulatorio Europa, Perugia - Olivia Morelli, Daniele Rosignoli, Elisabetta Schiaroli, Ospedale Santa Maria della Misericordia, Perugia - Cinzia Di Giuli, Ospedale S. Maria di Terni, Terni.

VENETO - Carla Manuppelli, Ospedale San Martino - Azienda ULSS 1, Belluno - Lorenzo Lomonaco, Ospedale Orlandi - ULSS 22, Bussolengo (VR) - Renato Marin, ULSS3 Serenissima Mirano, Mirano (VE) - Alessandro Vario, Ospedale Riuniti Padova Sud Madre Teresa di Calcutta - ULSS 17, Monselice (PD) - Paolo Bocus, Silvia Storato, Ospedale Sacro Cuore Don Calabria, Negrar (VR) - Francesco Paolo Russo, Luisa Benvegnù, Patrizia Pontisso, AO di Padova, Padova - Franco Capra, Ospedale Pederzoli, Peschiera del Garda - Antonio Carlotto, Roberto Ferretto, Ospedale Unico di Santorso - Azienda ULSS 4, Santorso (VI) - Marta Fiscon, ASL 3, Verona - Donatella Ieluzzi, Ospedale Borgo Trento, Verona - Franco Capra, Ospedale Borgo Roma, Verona - Vinicio Manfrin, Ospedale Vicenza - Azienda ULSS 6, Vicenza.

**SerD HCPs**

ABRUZZO - Rosa Grazia Costa, ASL Teramo - Giulianova, Giulianova (TE) - Paola Polci, ASL Teramo, Teramo.

BASILICATA - Niccolò Onorati, ASL Matera, Matera - Giuseppina Agriesti, SerD Potenza, Potenza.

CALABRIA - Vincenzo Mellace, ASL Catanzaro, Catanzaro - Roberto Calabria, Antonio Mastroianni, ASP Cosenza, Cosenza - Giuseppe Palucci, ASP Crotone, Crotone - Sergio Torchia, SerD Crotone, Crotone - Giuseppina Tufo, SerD Scalea Scalea (CS) - Carmela Maria Garista, ASP Reggio Calabria - Siderno Siderno (RC) - Giulia Audino, SerD Soverato, Soverato (CZ) - Anna Di Noia, ASP Cosenza - Trebisacce, Trebisacce (CS).

CAMPANIA - Luigi Stella, ASL Napoli 3 Sud, Napoli - Prisco Vicidomini, SerD 41 - Nocera Inferiore (Distr. 60), Nocera Inferiore (SA) - Domenico Cante, SerD 27 - Pozzuoli (Asl Na2 Nord - Distr.54), Pozzuoli (NA) - Patrizia Oliva, ASL Salerno, SerD 2, Salerno - Marina Di Matteo, ASL Napoli 3 Sud - Torre Annunziata, Torre Annunziata (NA).

EMILIA ROMAGNA - Claudio Comaschi, SerD Est - Bologna, Bologna - Salvatore Giancane, AUSL di Bologna, Bologna - Marco Viaggi, ASL di Bologna - Budrio, Budrio (BO) - Cristina Azzali, AUSL Parma, Parma - Sonja Aprile, Silvia Riccardi, SerD Parma, Parma - Matilde Bianchini, SerD Pavullo nel Frignano, Pavullo nel Frignano (MO) - Giovanni Greco, SerD Ravenna, Ravenna - Luana Oddi, AUSL Reggio nell’Emilia, Reggio Emilia - Maria Caterina Staccioli, AUSL della Romagna, Rimini.

FRIULI VENEZIA GIULIA - Elisabetta Savoini, SerD Azzano Decimo, Azzano Decimo (PN) - Antonio Natoli, ASS 5 “Friuli Occidentale”, Pordenone - Ariadna Baez, ASS 1 “Triestina”, Trieste - Roberta Balestra, SerD, Trieste - Hamid Kashanpour, Azienda Sanitaria Universitaria Integrata di Udine (ASUIUD), Udine - Tiziana Lombardelli, Dipartimento delle Dipendenze di Udine, Udine.

LAZIO - Giuseppe Barletta, SerD Asl Roma / F - Capena, Capena (RM) - Alessandro Ricci, Lucio Maciocia, ASL Frosinone - Cassino, Cassino (FR) - Cristiana Sarno, SerD Asl Roma / G - 06 Colleferro, Colleferro (RM) - Gianna Spaziani, ASL Frosinone, Frosinone - Emanuela Bernardini, ASL Roma 5 - Monterotondo, Monterotondo (RM) - Giovanni Palmieri, SerD Asl Roma / G - 05 Palestrina, Palestrina (RM) - Maria Antonietta Del Grosso, Marina Realacci, Letizia Rocchi, Rita Solli, ASL Roma 2, Roma - Pietro Casella, SerD Asl Roma / E - Municipio XVII, Roma - Donatella Vetrano, SerD, Roma - Maria Paola Giardino, ASL Latina - Terracina, Terracina (LT) - Massimo Persia, ASL Roma 5 - Tivoli - Villa Adriana, Tivoli (RM) - Anna Rita Giaccone, SerD 05 Viterbo, Viterbo.

LIGURIA - Javad Hossein Nejad, SerD Genova Centro-Levante - Distretto 13, Genova - Monica Arcellaschi, ASL 4 Chiavarese, Lavagna (GE) - Ina Hinnenthal, ASL 1 Imperiese, Imperia.

LOMBARDIA - Riccardo Mariano, SerD Appiano Gentile, Appiano Gentile (CO) - Fabrizio Cheli, Paolo Donadoni, ASST Papa Giovanni XXIII - Bergamo, Bergamo - Marco Riglietta, Dipartimento delle Dipendenze di Bergamo, Bergamo - Fabio Guerrini, SerD Corsico, Corsico (MI) - Antonio Francesco Prete, ASST di Crema, Crema (CR) - Damaris Rovida, ATS Brianza - Lecco, Lecco - Maurizio Mattioni Marchetti, SMI - Servizio Multidisciplinare Integrato Broletto, Lecco - Antonina Cardia, SerD Limbiate, Limbiate (MB) - Concettina Varango, ASST Lodi, Lodi - Ugo Calzolari, ASST Papa Giovanni XXIII - Lovere, Lovere (BG) - Caterina Maltempo, SerD Magenta, Magenta (MI) - Marco Degli Esposti, SerD Mantova, Mantova - Edoardo Cozzolino, ASST Fatebenefratelli Sacco, Milano - Antonella Di Marco, Letizia Testa, SerD 2 Piazzale Accursio - Milano, Milano - Paola Coppin, SerD 2 Conca del Naviglio - Milano, Milano - Attilio Cocchini, UO Carcere - Casa Circondariale di Monza, Monza - Cecilia Agnelli, ASST della Franciacorta - Orzinuovi, Orzinuovi (BS) - Enrico Coppola, Smi Aga - Servizio Multidisciplinare Integrato, Pontirolo Nuovo (BG) - Maurizio Parma, ASST della Franciacorta - Rovato, Rovato (BS) - Enza Barioglio, SerD S. Angelo Lodigiano, S. Angelo Lodigiano (LO) - Massimo Tarantola, SerD Sondrio, Sondrio - Claudio Tosetto, SerD Varese Nord, Varese - Cinzia Assi, SerD Vizzolo Predabissi, Vizzolo Predabissi (MI).

MARCHE - Marco Quercia, SerD, Ascoli Piceno - Giovanna Diotallevi, S.T.D.P. Pesaro, Pesaro - Manuela Falcinelli, Dipartimento Dipendenze Patologiche di Senigallia, Senigallia (AN).

PIEMONTE - Luigi Bartoletti, SerD 01 Alessandria, Alessandria - Valentina Zanoli, ASL di Novara, Arona (NO) - Daniele Pini, ASL Torino 3 - Beinasco, Beinasco (TO) - Lorenzo Somaini, SerD 01 Biella, Biella - Enrica Tapra, ASL Torino 4 - Ivrea, Ivrea - Alessandra Vallino, ASL Torino 4 - Ivrea, Ivrea (TO) - Liborio Martino Cammarata, ASL di Novara, Novara - Giovanni Pistone, Maria Enrica Rossi, ASL di Novara, Novara - Alessandro Barbero, SerD Trecate, Novara - Antonina Scarpinato, ASL Torino 3 - Rivoli, Rivoli (TO) - Ivana Conterno, ASL Cuneo 1 - Savigliano, Savigliano (CN) - Enrico De Vivo, Asl To2 Nord Torino - Distretto 5, Torino - Marina Bellinato, Sede distaccata Torino, Torino - Daniela Mussi, ASL Alessandria - Tortona, Tortona (AL) - Chiara Crosa Lenz, SerD 01 Verbania-Pallanza, Verbania.

PUGLIA - Francesca Calvario, ASL Bari - Altamura, Altamura (BA) - Gianfranco Mansi, SerD Andria, Andria (BT) - Fausto Campanozzi, SerD, Apricena (FG) - Giovanna Valentini, ASL Bari, Bari - Giuseppe Zizza, SerD Castellaneta, Castellaneta (TA) - Tommaso Del Giudice, Dipartimento delle Dipendenze di Foggia, Foggia - Margherita D’Ancona, SerD Francavilla Fontana, Francavilla Fontana (BR) - Vittorio Scrimieri, SerD Galatina, Galatina (LE) - Giuseppina Brizzi, SerD Giovinazzo, Giovinazzo (BA) - Maria De Finis, Angelo De Giorgi, ASL Foggia - Manfredonia, Manfredonia (FG) - Vincenza Ariano, ASL Taranto - Martina Franca, Martina Franca (TA) - Luigi Corvaglia, Eugenia Vernole, ASL Bari - Monopoli, Monopoli (BA) - Vito Santini, SerD San Severo, San Severo (FG).

SARDEGNA - Fabia Anna Ferri, ASL Cagliari, Cagliari - Rosalba Cicalò, ASL Nuoro, Nuoro.

SICILIA -

Paolo Castorina, SerD Adrano, Adrano (CT) - Giovanni Marrella, ASP Agrigento, Agrigento - Guido Faillace, ASP Trapani - Alcamo, Alcamo (TP) - Giuliano Milazzo, SerD Bagheria, Bagheria (PA) - Vito Lombardo, SerD Castelvetrano, Castelvetrano (TP) - Giuseppe Filippone, SerD Palermo 1, Palermo - Livia Scichilone, SerD S. Cataldo, San Cataldo (CL).

TOSCANA - Anna Maria Sbrilli, AUSL Toscana Sud Est - Abbadia San Salvatore, Abbadia San Salvatore (SI) - Daniele Pieralli, AUSL 8 di Arezzo, Arezzo - Giovanni Tavanti, AUSL 10 di Firenze - Bagno a Ripoli, Bagno a Ripoli (FI) - Paola Trotta, SerD Zona Ufm 1 - S.E, Bagno a Ripoli (FI) - Marina Carletti, SerD Zona Ufm 2 - S.E., Figline e Incisa Valdarno (FI) - Francesca Fabrizi, SerD Zona Ufm B - Serd 3 Fi, Firenze - Adriana Iozzi, SerD Zona Ufm C - Quartiere 5 - Fi Centro, Firenze - Lucia Giannini, AUSL Toscana Sud Est - Montepulciano, Montepulciano (SI) - Marco Baldi, AUSL Toscana Sud Est - Sansepolcro, Sansepolcro (AR).

TRENTINO ALTO ADIGE - Ilaria Bracardi, Bettina Meraner, AS dell’Alto Adige, Bolzano - Laura Menapace, APSS Trento - Distretto Centro-sud - Rovereto, Rovereto (TN) - Annora Ratti, APSS Trento, Trento.

UMBRIA - Giuliano Dozzini, Dipartimento delle Dipendenze di Foligno, Foligno (PG) - Daniela Gallucci, Roberta Gaudenzi, SerD Perugia, Perugia - Roberto Cruciani, SerD Spoleto, Spoleto (PG) - Marco Cuccuini, Mara Gilioni, Valeria Morbiducci, SerD Terni, Terni.

VENETO - Raimondo Alonge, ULSS 1 Dolomiti - Belluno, Belluno - Caterina Peron, Alessandro Rovea, AULSS 6 Euganea - Camposampiero, Camposampiero (PD) - Roberto Manera, AULSS 2 Marca Trevigiana - Castelfranco Veneto, Castelfranco Veneto (TV) - Barbara Salmaso, AULSS 3 Serenissima - Chioggia, Chioggia (VE) - Laura Suardi, AULSS 3 Serenissima - Dolo, Dolo (VE) - Giancarlo Zecchinato, SerD Este, Este (PD) - Damiano Barbiero, SerD Cittadella, Este (PD) - Novella Ghezzo, Azienda ULSS 3 Serenissima, Mestre (VE) - Nicola Gentile, SerD Mirano, Mirano (VE) - Rosalia Anna Lo Cascio, AULSS 8 Berica - Montecchio Maggiore, Montecchio Maggiore (VI) - Daria Monteforte, SerD 2 - Servizio Territoriale per le Dipendenze - Montecchio Maggiore, Montecchio Maggiore (VI) - Francesco Antonioli, Giuseppina Certa, SerD Noventa, Noventa Vicentina (VI) - Mauro Codogno, Giovanna Mantovani, SerD Noventa, Noventa Vicentina (VI) - Salvatore Lobello, AULSS 6 Euganea, Padova - Diego Saccon, SerD San Dona’ di Piave, San Dona’ di Piave (VE) - Maria Cristina Fanton, SerD 2 - Servizio Territoriale per le Dipendenze - Valdagno, Valdagno (VI) - Enrica Milan, Alessandro Pani, AULSS 3 Serenissima, Venezia - Eva D’Incecco, SerD Rio Novo Dorsoduro, Venezia.
